# Supplementary material for: Effects of increasing molybdenum under low nitrogen input on yield, nitrogen-metabolizing enzymes and nitrogen use efficiency of winter wheat cultivars
Source: PLoS One. 2026 Mar 5;21(3):e0339840. doi: 10.1371/journal.pone.0339840 (PMC12962463; doi:10.1371/journal.pone.0339840)
Supplement: Attachment — Submitted filename: PONE-D-25-09819 (2).pdf [file pone.0339840.s002.pdf]

PLOS ONE

Effects of increasing molybdenum under low nitrogen input on yield, nitrogen-metabolizing enzymes and nitrogen use efficiency of winter wheat cultivars

--Manuscript Draft--

**Manuscript Number:**	PONE-D-25-09819
**Article Type:**	Research Article
**Full Title:**	Effects of increasing molybdenum under low nitrogen input on yield, nitrogen-metabolizing enzymes and nitrogen use efficiency of winter wheat cultivars
**Short Title:**	Low Nitrogen and Molybdenum Effects on Winter Wheat Yield and Nitrogen Metabolism
**Corresponding Author:**	Youning Ning Wang<br>Hubei Engineering University<br>Xiaogan, CHINA
**Keywords:**	molybdenum application rate; yield; wheat; nitrogen use efficiency; nitrogen metabolizing enzymes
**Abstract:**	In wheat production, improving resource utilization and grain yield is the goal researcher have been pursuing. This study aimed to investigate if a regulated nitrogen (N) and molybdenum (Mo) fertilizer management could increase wheat yield and N use efficiency (NUE). This study reports theeffects of three N application levels (N0: 0 kg N ha−1, N1: 75 kg N ha−1, N2: 150 kg N ha−1) and a combination of three Se application levels (Mo1: 0 kg Na2MoO4 ha−1, Mo2: 0.75 kg Na2MoO4 ha−1, Mo3: 1.5 kg Na2MoO4 ha−1) on N metabolism, NUE and yield in wheat. The results showed that average grain yield increases were 61.78% under the different N input, and 11.71% under the Mo input. Nitrogen absorption efficiency (NAE), nitrogen recovery efficiency (NRE) and partial factor productivity (PEPN) significantly (P<0.01) increased low N rates (N1) but significantly (P<0.05) decreased with at high N rates (N2). NAE and PEPN significantly (P<0.01) different under different Mo input. NAE significantly decreased with the increased of Mo input. Conversely, the PEPN increased with the increased of Mo input, the N1+Mo3 had the higher PEPN.With the increase of Mo, the leaf area index and net photosynthetic rate increased significantly at all growth stages. Compared with Mo1, the leaf area index (LAI) of Mo2 and Mo3 increased by 13.68% and 19.33%, respectively, and the photosynthetic rate increased by 26.47% and 45.07%. Compare with Mo0, NR, GS and GOGAT increased by 1.15%, 4.60%, and 1.95% in N1 and by 3.80%, 7.31%, and 4.94% in N2, respectively. We conclude that high Mo and low nitrogen input could prolong the duration of green leaf, improve the activity of nitrogen metabolism enzyme in middle and late growth, and improve the wheat spikelets per panicle and N metabolism and has the potential to increase wheat yield and NUE.
**Order of Authors:**	Di Yang
	Qixia Wu
	Youning Ning Wang
**Additional Information:**	
**Question**	**Response**
**Financial Disclosure**<br><br>Enter a financial disclosure statement that describes the sources of funding for the work included in this submission. Review the submission guidelines for detailed requirements. View published research articles from *PLOS ONE* for specific examples.	Yes

Powered by Editorial Manager® and ProduXion Manager® from Aries Systems Corporation

This statement is required for submission and **will appear in the published article** if the submission is accepted. Please make sure it is accurate.<br><br>**Funded studies**<br>Enter a statement with the following details:<br>• Initials of the authors who received each award<br>• Grant numbers awarded to each author<br>• The full name of each funder<br>• URL of each funder website<br>• Did the sponsors or funders play any role in the study design, data collection and analysis, decision to publish, or preparation of the manuscript?<br><br>Did you receive funding for this work?	
Please add funding details.<br>as follow-up to "**Financial Disclosure**<br><br>Enter a financial disclosure statement that describes the sources of funding for the work included in this submission. Review the submission guidelines for detailed requirements. View published research articles from *PLOS ONE* for specific examples.<br><br>This statement is required for submission and **will appear in the published article** if the submission is accepted. Please make sure it is accurate.<br><br>**Funded studies**<br>Enter a statement with the following details:<br>• Initials of the authors who received each award<br>• Grant numbers awarded to each author<br>• The full name of each funder<br>• URL of each funder website<br>• Did the sponsors or funders play any role in the study design, data collection and analysis, decision to publish, or preparation of the manuscript?	This work was supported by the Natural Science Funds of Hubei Province of China(2024AFB1015).

Powered by Editorial Manager® and ProduXion Manager® from Aries Systems Corporation

Did you receive funding for this work?"	
Please select the country of your main research funder (please select carefully as in some cases this is used in fee calculation).<br>as follow-up to "**Financial Disclosure**<br><br>Enter a financial disclosure statement that describes the sources of funding for the work included in this submission. Review the submission guidelines for detailed requirements. View published research articles from *PLOS ONE* for specific examples.<br><br>This statement is required for submission and **will appear in the published article** if the submission is accepted. Please make sure it is accurate.<br><br>**Funded studies**<br>Enter a statement with the following details:<br>• Initials of the authors who received each award<br>• Grant numbers awarded to each author<br>• The full name of each funder<br>• URL of each funder website<br>• Did the sponsors or funders play any role in the study design, data collection and analysis, decision to publish, or preparation of the manuscript?<br><br>Did you receive funding for this work?"	CHINA - CN
**Competing Interests**<br><br>Use the instructions below to enter a competing interest statement for this submission. On behalf of all authors, disclose any competing interests that could be perceived to bias this work—acknowledging all financial support and any other relevant financial or non-financial competing interests.<br><br>This statement is required for submission	The authors have declared that no competing interests exist.

and **will appear in the published article** if the submission is accepted. Please make sure it is accurate and that any funding sources listed in your Funding Information later in the submission form are also declared in your Financial Disclosure statement.<br><br>View published research articles from *PLOS ONE* for specific examples.<br><br>**NO authors have competing interests**<br><br>Enter: *The authors have declared that no competing interests exist.*<br><br>**Authors with competing interests**<br><br>Enter competing interest details beginning with this statement:<br><br>*I have read the journal's policy and the authors of this manuscript have the following competing interests: [insert competing interests here]*<br><br>* typeset	
**Ethics Statement**<br><br>Enter an ethics statement for this submission. This statement is required if the study involved:<br><br>• Human participants<br>• Human specimens or tissue<br>• Vertebrate animals or cephalopods<br>• Vertebrate embryos or tissues<br>• Field research<br><br>Write "N/A" if the submission does not require an ethics statement.<br><br>General guidance is provided below. Consult the submission guidelines for detailed instructions. **Make sure that all information entered here is included in the Methods section of the manuscript.**	N/A

**Format for specific study types**<br><br>**Human Subject Research (involving human participants and/or tissue)**<br>• Give the name of the institutional review board or ethics committee that approved the study<br>• Include the approval number and/or a statement indicating approval of this research<br>• Indicate the form of consent obtained (written/oral) or the reason that consent was not obtained (e.g. the data were analyzed anonymously)<br><br>**Animal Research (involving vertebrate animals, embryos or tissues)**<br>• Provide the name of the Institutional Animal Care and Use Committee (IACUC) or other relevant ethics board that reviewed the study protocol, and indicate whether they approved this research or granted a formal waiver of ethical approval<br>• Include an approval number if one was obtained<br>• If the study involved *non-human primates*, add *additional details* about animal welfare and steps taken to ameliorate suffering<br>• If anesthesia, euthanasia, or any kind of animal sacrifice is part of the study, include briefly which substances and/or methods were applied<br><br>**Field Research**<br><br>Include the following details if this study involves the collection of plant, animal, or other materials from a natural setting:<br>• Field permit number<br>• Name of the institution or relevant body that granted permission	
**Data Availability**<br><br>Authors are required to make all data underlying the findings described fully available, without restriction, and from the time of publication. PLOS allows rare exceptions to address legal and ethical concerns. See the PLOS Data Policy and FAQ for detailed information.	Yes - all data are fully available without restriction

A Data Availability Statement describing where the data can be found is required at submission. Your answers to this question constitute the Data Availability Statement and **will be published in the article**, if accepted.<br><br>**Important:** Stating 'data available on request from the author' is not sufficient. If your data are only available upon request, select 'No' for the first question and explain your exceptional situation in the text box.<br><br>Do the authors confirm that all data underlying the findings described in their manuscript are fully available without restriction?	
**Describe where the data may be found in full sentences. If you are copying our sample text, replace any instances of XXX with the appropriate details.**<br><br>• If the data are **held or will be held in a public repository**, include URLs, accession numbers or DOIs. If this information will only be available after acceptance, indicate this by ticking the box below. For example: *All XXX files are available from the XXX database (accession number(s) XXX, XXX.*).<br>• If the data are all contained **within the manuscript and/or Supporting Information files**, enter the following: *All relevant data are within the manuscript and its Supporting Information files.*<br>• If neither of these applies but you are able to provide **details of access elsewhere**, with or without limitations, please do so. For example:<br><br>*Data cannot be shared publicly because of [XXX]. Data are available from the XXX Institutional Data Access / Ethics Committee (contact via XXX) for researchers who meet the criteria for access to confidential data.*<br><br>*The data underlying the results presented in the study are available from (include the name of the third party*	All relevant data are within the manuscript and its Supporting Information files.

and contact information or URL).<br>• This text is appropriate if the data are owned by a third party and authors do not have permission to share the data.<br><br>* typeset	
Additional data availability information:	

Cover Letter Click here to access/download;Manuscript;plos one.docx

Effects of increasing molybdenum under low nitrogen input on yield, nitrogen-metabolizing enzymes and nitrogen use efficiency of winter wheat cultivars

Di Yang[1,2], Qixia Wu[2],and Youning Wang[1,*]

[1] Hubei Key Laboratory of Quality Control of Characteristic Fruits and Vegetables, Hubei Engineering University, Xiaogan 432000, China; yd84893104@163.com (D.Y.);
[2] College of Agriculture, Yangtze University, Jingzhou 434025, China
* Correspondence: king_union@126.com (Y.-n.W.)

Abstract

In wheat production, improving resource utilization and grain yield is the goal researcher have been pursuing. This study aimed to investigate if a regulated nitrogen (N) and molybdenum (Mo) fertilizer management could increase wheat yield and N use efficiency (NUE). This study reports theeffects of three N application levels (N0: 0 kg N ha^{-1}, N1: 75 kg N ha^{-1}, N2: 150 kg N ha^{-1}) and a combination of three Se application levels (Mo1: 0 kg Na_2MoO_4 ha^{-1}, Mo2: 0.75 kg Na_2MoO_4 ha^{-1}, Mo3: 1.5 kg Na_2MoO_4 ha^{-1}) on N metabolism, NUE and yield in wheat. The results showed that average grain yield increases were 61.78% under the different N input, and 11.71% under the Mo input. Nitrogen absorption efficiency (NAE), nitrogen recovery efficiency (NRE) and partial factor productivity (PEPN) significantly (P<0.01) increased low N rates (N1) but significantly (P<0.05) decreased with at high N rates (N2). NAE and PEPN significantly (P<0.01) different under different Mo input. NAE significantly decreased with the increased of Mo input. Conversely, the PEPN increased with the increased of Mo input, the N1+Mo3 had the higher PEPN.With the increase of Mo, the leaf area index and net photosynthetic rate increased significantly at all growth stages. Compared with Mo1, the leaf area index (LAI) of Mo2 and Mo3 increased by 13.68% and 19.33%, respectively, and the photosynthetic rate increased by 26.47% and 45.07%. Compare with Mo0, NR, GS and GOGAT increased by 1.15%, 4.60%, and 1.95% in N1 and by 3.80%, 7.31%, and 4.94% in N2, respectively. We conclude that high Mo and low nitrogen input could prolong the duration of green leaf, improve the activity of nitrogen metabolism enzyme in middle and late growth, and improve the wheat spikelets per panicle and N metabolism and has the potential to increase wheat yield and NUE.

Introduction

Wheat one of the most important crops worldwide and provides nutritive food to more than one-third of the global population, and plays a pivotal role in food industry[1]. It is our national condition to have a large population and less land. The fundamental way to solve our food security and meet people's increasing demand for wheat products is to constantly increase the yield of wheat[2].

Molybdenum is a trace element in soil. The content of molybdenum in soil of countries around the world is generally 0.5~5.0 mg/kg, and the average content is 2.3 mg/kg[3]. Molybdenum deficiency is common in soils worldwide, especially in acidic soils[4]. Despite the small demand of plants for molybdenum, molybdenum deficiency has been reported in more than 40 species of higher plants worldwide[5]. The leaf color of wheat seedlings was

white when molybdenum was deficient, and severe necrosis occurred from the tip along the leaf direction[6]. Degreenization and yellowing occurred at the edge of wheat old leaves when molybdenum was deficient[7]. When soil available Mo level was insufficient, wheat dry matter accumulation was reduced, tillering was blocked, growth was slowed, and flowering was delayed[8]. Application of molybdenum fertilizer can increase tillering number of wheat, accelerate wheat growth rate, and promote early maturation of wheat[7]. Mo application can improve seedling vigor and promote growth and development of wheat[5]. Application of molybdenum fertilizer can improve the stability and integrity of organelles and membrane structure of winter wheat leaf cell, enhance cold resistance of winter wheat, and promote growth and development of winter wheat[9-10]. Molybdenum had significant effects on the nodulation and growth of artificially induced wheat[11]. With the increase of molybdenum application amount, wheat dry matter weight showed a trend of first increasing and then decreasing. High molybdenum content in wheat seeds can reduce the amount of molybdenum fertilizer applied in acidic soils. Hu et al.[12] found that molybdenum promoted the distribution of nitrogen absorbed by winter wheat to the growth center at jointing and heading stages, and nitrate reduction was more intense under balanced molybdenum and nitrogen supply. Therefore, appropriate amount of molybdenum fertilizer can be used to regulate the transport and distribution of nitrogen in plants.

The average nitrogen application amount of wheat in the North China plain had reached 424 kg ha^{-1}, which was much higher than the nutrient consumption in the same period[13-14]. At present, many experts have conducted studies on reduced nitrogen application. Liang et al.[15] found that there was no significant change in corn yield after reduced nitrogen application by 15%, but the highest yield was obtained by reducing nitrogen by 25%. At present, the nitrogen utilization rate of wheat production in China is only 21.2%~35.9%, which is much lower than the foreign level of 40%~60%[16]. Rational reduction of nitrogen fertilizer has become a hot topic in wheat fertilization research. Previous studies have shown that appropriate nitrogen reduction based on the amount of nitrogen applied by farmers will not reduce wheat yield[17-18].

Within a certain range of nitrogen application rate, wheat grain yield increased with the increase of nitrogen application rate, and yield decreased with high nitrogen application rate[19]. However, the current research and application of varieties mainly concentrated on dry stubble strong and medium gluten wheat and rice stubble medium and weak gluten wheat, it is believed that reasonable fertilization can promote the simultaneous increase of wheat grain yield and protein[20-22]. The suitable nitrogen application amount varies with regional ecological conditions and soil fertility, and the target yield and quality index requirements are also different[23]. Some other studies believed that the increase of nitrogen application rate could increase the panicle number, spikelet per panicle and wet gluten content of wheat, but too high nitrogen application rate would lead to the decrease of crude protein content and precipitation value of weak gluten wheat in dry stubble[24]. The biological yield of wheat was increased by increasing soil N level and Mo level. Although the biological yield of wheat could be increased significantly with only nitrogen fertilizer application, the economic coefficient decreased. After molybdenum application, the economic coefficient of wheat was significantly improved, dry matter could be fully utilized, and dry matter transfer and accumulation in wheat body to harvest site could be promoted, so as to obtain high yield[7].

In this study, field experiments were conducted using two wheat varieties grown at different N application and Mo rates and planting densities. We used the method of cluster analysis, and the different agronomic characters were analyzed by variance analysis. The objectives of this study were: 1) to compare grain yield, NUE and nitrogen-metabolizing enzymes under different N and Mo input rates, and 2) to identify the effects of the interaction between N and Mo on N metabolism, N use efficiency and yield in wheat.

Materials and Methods

Planting and Cultivation Management of Wheat

Pot experiments were conducted in the experimental farm at Yangtze University (latitude: 30°23'; longitude: 112°29′), Jingzhou, in 2022. The soil samples from the field's 0~30 cm plough layer in the experimental area before sowing were analyzed.

The soil was calcareous alluvial with pH 6.5, 13.7 g kg^{-1} organic matter, 617.2 mg kg^{-1} alkali-hydrolysable N, 14.5 mg kg^{-1} available P, and 94.2 mg kg^{-1} available K. Then, 2.3 kg of sieved dry soil containing 1.5 g of compound fertilizer (N + P_2O_5 + K_2O ≥ 48%), and 30.0 g of organic fertilizer was mixed evenly for each pot (28 cm high, 13 cm in diameter).

Five wheat seeds were sown on 17 October 2021, and three seedlings with similar plant sizes were retained at the three-leaf stage. Each pot was top-dressed with 0.2 g of urea at the middle-tillering stage. Crop management followed standard cultural practices. Insects were intensively controlled with pesticides to avoid biomass and yield losses.

Experimental Design

Research manuscripts reporting large datasets that are deposited in a publicly available database should specify where the data have been deposited and provide the relevant accession numbers. If the accession numbers have not yet been obtained at the time of submission, please state that they will be provided during review. They must be provided prior to publication.

Sampling and Measurement

Grain yield and yield components

Panicle number was measured in 3 unsampled pots at wheat maturation stage, and the number of grains per ear and 1000-grain weight of 30 wheat ears were investigated randomly. After all the wheat ears were cut manually, they were threshed by a small seed thresher, dried and weighed, and the grain yield with water content of 13% was calculated.

Leaf area index and Photosynthetic Capacity

At 7, 14, 21 and 28 days after flowering, 15 plants with uniform growth were selected for each treatment. The LAI was determined by CI-203 leaf area instrument (US CID).

The photosynthetic rate was determined by Li-6800 portable photosynthesis measurement system (Li-Cor), using open gas path, CO_2 concentration was about 3μmol L^{-1}, red and blue light source leaf chamber was selected. The photosynthetically active radiation (PAR) was set as 1000 μmol m^{-2} s^{-1}. The measurement time was 9:00-11:00 in the morning.

Dry Matter Accumulation and nitrogen use efficiency

At 7d, 14d, 21d and 28d after flowering, 6-point plants with uniform growth were selected for each treatment, washed and defoliated at 105℃ for 20 min, dried at 80℃ to constant mass before weighing, and then powdered. Samples of the crushed dry matter were taken, weighed at 0.5g for each sample and placed in a Kjeldahl flask, followed by adding 0.3g of potassium sulphate-copper sulphate contact agent. Concentrated Sulfuric acid 2.0mL, digestion furnace digestion. The nitrogen content was measured by automatic Kjeldahl nitrometer and nitrogen uptake was calculated.

Nitrogen agronomic efficiency (NAE) was calculated by the following formula: NAE (kg kg^{-1}) = [(grain yield with N treatments - grain yield without N) / amount of N fertilizer applied]. Nitrogen recovery efficiency (NRE) was calculated by the following formula: NRE (%) = [(sum of N content in all aboveground components under N treatment - sum of N content in all aboveground components without N) / amount of N fertilizer applied]. Partial

factor productivity (PFPN) of applied N was calculated by the following formula: N fertilizer (PFPN, kg kg^{-1}) = [grain yield / amount of N fertilizer applied].

Determination of the nitrogen-metabolizing enzymes

The nitrate reductase (NR, EC 1.6.6.1) activity assay followed the method proposed by Wu et al[14]. We mixed 1.3 mM EDTA and 10 mM cysteine to obtain 4 mL of cold 25 mM sodium phosphate (pH 8.7) buffer. The frozen plant leaf samples were ground into a homogenate in the buffer, which was centrifuged at 4000×g rpm and 4 ℃for 30 min. The centrifugation supernatant, 0.1 M KNO_3 and 2.82 mM NADH formed the reaction solution. We added NADH to start the reaction, then incubated the mixture for 30 min. The reaction was ended with 1% sulfanilamide and 0.02% N-phenyl-2-naphthylamine and left for 15 min. After centrifugation at 4000× g rpm for 5 min, the absorbance was determined at 540 nm.

The glutamine synthetase (GS) activity was determined according to the methods of (Hou et al., 2019; Zhang et al., 2022). Fresh leaves (1.0 g) were homogenized in 3.0 ml of Tris-HCl (0.05 M, pH 8.0) in a pre-cooled mortar in an ice bath, followed by extraction with $MgSO_4$ (2 mM), DTT (2 mM) and sucrose (0.4 M). The homogenate was centrifuged at 15000 rpm at 4 ℃for 20 min. An aliquot of the crude enzyme solution (0.7 ml) and 0.7 ml of 40 mM ATP were added to 1.6 ml of reaction mixture B containing imidazole-HCl buffer (0.1 M, pH 7.4), sodium hydrogen glutamate (20 mM), $MgSO_4$ (80 mM), Cysteine (20 mM), EDTA (2 mM) and hydroxylamine hydrochloride (80 M). For the control, reaction mixture A without hydroxylamine hydrochloride was used instead of reaction mixture B. The mixture was incubated at 25 ℃for 15 min. Approximately 1 ml of chromogenic agent (TCA (0.2 M), FeCl3 (0.37 M), HCl (0.6 M)) was added to terminate the reaction. After centrifugation at 5000 rpm for 10 min, the absorbance of the supernatant was measured at 540 nm.

The glutamate synthase (GOGAT) activity was determined according to the methods of (Hou et al., 2019; Zhang et al., 2022). Fresh leaves (0.4 g) were ground in a chilled mortar in 25 mmol/L Tris-HCL buffer (pH 7.6) and the homogenate was centrifuged at 13000 rpm/min for 25 min. The GOGAT reaction mixture consisted of 0.4 ml 20 mmol/L L-glutamine, 0.5 ml 20 mmol/Lα-ketoglutarate, 0.1 ml 10 mmol/L KCl, 0.2 ml 3 mmol/L NADH and 0.3 ml enzyme extract. Through monitoring the oxidation of NADH, GOGAT activity was assayed at 340 nm using a 752UV-Vis spectrophotometer.

Data Analysis

Data were analyzed using three-way analysis of variance with R 4.3.1 (Analytical Software, Tallahassee, FL, USA). The means were compared between N treatments and rice cultivars based on the least significant difference (LSD) test at a 0.05 probability level.

Results

Yield and yield components

N and Mo application rate significantly ($P<0.01$) affected grain yield (Figure 1). Grian yield significantly increased with the increasing of N and Mo input. Average grain yield increases were 61.78% under the different N input, and 11.71% under the Mo input. Grain yield of N1+Mo2 and N1+Mo3 was 6249.9 and 6394.0 kg ha^{-1}, which was 0.68% and 3.0% higher than N2+Mo1, respectively.

Yield component responses to plant density varied with N application rate (Figure 1). Panicle number significantly ($P<0.05$) increased with N application rate. Panicle number

in the treatments receiving N was 73.9% and 99.3% higher, respectively, relative to the N0 treatment, but had not siginificantly under the Mo input. Spikelets per panicle significant (P<0.01) different under different N and Mo application rate. Spikelets per panicle significantly increased (N1) and either remained constant (N2) or decreased (N3) with increasing N application rate. With the increasing of Mo input, the spikelets per panicle of Mo2 and Mo3 were 5.02% and 7.62% higher than Mo1, respectively. The most spikelets per panicle was 46.6, which was under N1+Mo3 treatment. The effects of Mo application rate on 1000-grain weight were significant different (P<0.05). Average 1000-grain weight in Mo3 increases were 4.41% higher than Mo1.

Leaf area index and photosynthetic rate

N and Mo application rate significantly (P<0.01) affected LAI (Figure 2). LAI in the treatments receiving N was 4.83% higher, respectively, relative to the N0 treatment. With the passage of time, LAI after flowering was significantly decreased under different nitrogen treatments, and was steadily decreased from 7 to 14 days after flowering, but was exponentially decreased from 14 to 28 days after flowering. With the increase of Mo, the leaf area index increased significantly at all growth stages, and Mo2 and Mo3 increased by 13.68% and 19.33% compared with Mo1, respectively.

Photosynthetic rate significantly (P<0.01) different under different Mo input (Figure 3). LAI in Mo2 and Mo3 were 26.47% and 45.07% higher, respectively, relative to the Mo0 treatment. The photosynthetic rate significantly decreased with the passage of growth period, similar to LAI, which decreased steadily from 7-14d and exponentially from 14-28d.

Total dry weight, nitrogen accumulation, and NUE

Total dry weight at maturity significantly (P<0.05) increased with N and Mo application rate (Tables 2). TDW in N1 and N2 were 109.45% and 137.22% higher than N0. TDW significantly increased (N1+Mo3) and either remained constant (N2+Mo1/Mo2) or lightly increased (N3) with increasing N and Mo. In contrast, N accumulation significantly increased under different N input, 93.44% and 156.35% higher in N1 and N2 than NO, respectively. The effects of Mo application rate on N accumulation were not significant (P>0.05).

NUE varied among treatments (Tables 2). N1 showed higher NAE, NRE, and PFP than N2. NAE, NRE and PFP significantly (P<0.01) increased low N rates (N1) but significantly (P<0.05) decreased with at high N rates (N2). Average NAE were 6.13% and 7.13% higher in the two wheat varieties, 2.24% and 10.62% in NRE, and 19.63%, 20.03% in PEPN under the N1 treatment relative to N2. NAE and PEPN significantly (P<0.01) different under different Mo input. NAE significantly decreased with the increased of Mo input. Conversely, the PEPN increased with the increased of Mo input, the N1+Mo3 had the higher PEPN.

Nitrogen metabolizing enzymes

N application significantly (P<0.01) increased N-metabolizing enzyme activity (Figure 4). Compare with N0, NR, GS and GOGAT increased by 8.94%, 25.95%, and 29.48% in N1 and by 21.92%, 64.21%, and 29.88% in N2, respectively. N-metabolizing enzyme activity significantly improved under different Mo input. Compare with Mo0, NR, GS and GOGAT increased by 1.15%, 4.60%, and 1.95% in N1 and by 3.80%, 7.31%, and 4.94% in N2, respectively. As time went on, N-metabolizing enzyme activity decreased significantly under different nitrogen treatments, stably at 7-14d after anthesis, and exponentially at 14-28d after anthesis.

Correlation analyses of grain yield, yield components, NUE, and nitrogen metabolizing enzymes at various nitrogen and Mo application rates

Correlation matrices among the various grain yield components, NUE parameters, N and Mo application rates are shown in Figure 5. N application rate significantly ($P < 0.01$) and positively correlated with yield, TDW, PN, NR, Naccu, LAI, GOGAT, NRE, and GS. Mo application rate significantly ($P < 0.05$) and positively correlated with GW and Pnco. Additionally, there was a significant ($P < 0.01$) positive correlation between yield and SP, Pnco, GS, Naccu, LAI, NRE, PEPN, GOGAT, TDW, PN, and NR.

Discussion

The yield of wheat is mainly affected by three factors: panicle number, spikelet per panicle and grain weight. Molybdenum plays an irreplaceable role in biological nitrogen fixation and nitric acid reduction[12,25]. Molybdenum is one of the essential trace elements in plants, and the nutritional relationship between molybdenum and nitrogen has attracted great attention[26-27]. The application of molybdenum fertilizer could eliminate the wheat yield caused by high nitrogen application rate and maintain the wheat yield, and whether urea or ammonium nitrate was used as the nitrogen source, molybdenum fertilizer could increase the wheat yield[28]. The pot experiment results of Du et al.[7] showed that the yield increase of winter wheat was not stable when molybdenum fertilizer was applied under high nitrogen fertilizer dosage, and nitrogen fertilizer dosage was one of the conditions for molybdenum fertilizer application in winter wheat[29]. We found that both yield and yield components increased with increasing N and Mo application levels, which is consistent with previous conclusions. Under the same N application, with the increase of Mo input, the increase of yield mainly came from the increase of spikelets per panicle. With the increasing of Mo input, the spikelets per panicle of Mo2 and Mo3 were 5.02% and 7.62% higher than Mo1, respectively. The most spikelets per panicle was 46.6, which was under N1+Mo3 treatment. The effects of Mo application rate on 1000-grain weight were significant different ($P<0.05$). Average 1000-grain weight in Mo3 increases were 4.41% higher than Mo1. while panicle number was not significantly different under different Mo input. In this study, grain yield of N1+Mo2 and N1+Mo3 was 6249.9 and 6394.0 kg ha^{-1}, which was 0.68% and 3.0% higher than N2+Mo1, respectively. N1+Mo2 and N1+Mo3 showed no difference from N3+M1, indicating that Mo has the potential to reduce the N input and compensate for the yield decline.

Nitrogen is an important element to ensure the high yield of wheat[30]. At present, in order to maintain a high yield, the application rate of nitrogen fertilizer has been maintained at a high level, which is the main reason for the low utilization rate of nitrogen fertilizer[31]. Tian et al.[32] showed that, nitrogen reduction affected the nitrogen uptake by white gluten wheat plants in the dry stubble half winter under the condition of low soil fertility, and the nitrogen apparent use efficiency decreased, but the nitrogen partial productivity and nitrogen physiological efficiency increased, while the nitrogen harvest index was little affected. In our study, NAE, NRE and PFP significantly ($P<0.01$) increased low N rates (N1) but significantly ($P<0.05$) decreased with at high N rates (N2). NAE significantly decreased with the increased of Mo input. Conversely, the PEPN increased with the increased of Mo input, the N1+Mo3 had the higher PEPN. This study showed excessively increasing the amount of N fertilizer would reduce NAE and NRE, which is consistent with the results of most studies[31-32]. Rational nitrogen fertilizer application is of great significance in improving wheat NUE. PEPN significantly improved under the Mo application rates, and it is significantly increased under low nitrogen (N1) condition, indicating that increasing Mo under low nitrogen (N1) treatment can effectively improve wheat nitrogen use efficiency.

Lack of molybdenum will inhibit the synthesis of photosynthetic pigments and lead to yellowing of plant leaves[33-34]. Appropriate amount of molybdenum is beneficial to plant

growth, while excessive molybdenum will lead to crop yield reduction and agricultural product quality decline[27]. Molybdenum application promoted the photosynthesis of winter wheat[34]. Under the condition of soil molybdenum pollution, the chlorophyll content of winter wheat decreased and stomatal restriction led to the decrease of photosynthetic rate, which affected the photosynthetic capacity of winter wheat, and finally led to the significant reduction of biological yield[35]. This is similar to the results of this study. Increasing Mo input significantly increased the LAI and photosynthetic rate at each growth stage, Mo could significantly prolong the duration of green leaf, improve the net photosynthetic rate at the later growth stage, promote grain filling and fruit setting, and increase yield.

NR plays an important role in plant regulation and nitrogen assimilation, and N and protein concentrations in leaves are related to their activities, while NR, GS and GOGAT play a key role in nitrogen metabolism through synergistic interaction with N[36]. In this study, appropriate nitrogen and molybdenum applications significantly increased N-metabolizing enzyme activity. Relevant studies showed that molybdenum application reduced the nitrate nitrogen content of winter wheat varieties[35], promoted the conversion of inorganic nitrogen to organic nitrogen, and provided more nitrogen sources for the synthesis of substances in the subsequent metabolic chain. At the same time, it could avoid the possible poisoning of plants caused by excessive accumulation of nitrate nitrogen[34-35]. This is similar to the results of this study, molybdenum application can effectively improve nitrogen metabolic pathway, enhance N-metabolizing enzyme activity, extend nitrogen metabolic intensity in later growth period, and improve nitrogen use efficiency, so as to achieve increased production and efficiency.

Conclusions

This study evaluated the contribution of photosynthetic characteristics, nitrogen utilization rate and nitrogen metabolic enzymes to yield under nitrogen reduction and molybdenum improvement. The results showed that increasing N and Mo input could significantly increase the yield, while increasing Mo under low nitrogen (N1) treatment could effectively compensate for the yield loss, even exceeding that under high nitrogen (N2+Mo1) treatment. The increase in yield was due to the increase in grain number per panicle and TDW due to the higher NRE, PEPN, LAI after flowering, photosynthetic rate and GOGAT under the increased Mo treatment. Therefore, increasing Mo input in reducing nitrogen application rates could effectively reduce yield loss and improve NUE. These findings bear important implications for achieving high yield and high efficiency in nutrient uptake and utilization in modern rice-production systems.

Supplementary Materials: not applicable.

Author Contributions: The contributors are Y.-n.W. for conceptualization and methodology; Q.-x.W. and D.-Y. for formal analysis and investigation/writing—original draft/supervision; D.-Y. for visualization, and writing—review/editing. All authors have read and agreed to the published version of the manuscript.

Funding: This work was supported by the Natural Science Funds of Hubei Province of China(2024AFB1015).

Data Availability Statement: The datasets used and/or analysed during the current study are available from the corresponding author on reasonable request.

Acknowledgments: I am very grateful to Yangtze University for providing experimental conditions

Conflicts of Interest: The authors declare that they have no competing interests.

References

1. Xu J J, Gao Z Y, Liu S, Elwafa S F, Tian H. A multienvironmental evaluation of the N, P and K use efficiency of a large wheat diversity panel. Field Crops Research, 2022, 286, 108634.
2. Hou S B, Dang H Y, Huang T M, Huang Q N, Li C, Li X H, Sun Y Y, Chu H X, Qiu W H, Liu J S, Shi M, He G, Siddique K H, Wang Z H. Targeting high nutrient efficiency to reduce fertilizer input in wheat production of China. Field Crops Research, 2023, 292, 108809.
3. Anke M, Seifert M. The biological and toxicological importance of molybdenum in the environment and in the nutrition of plants, animals and man. Part 1: Molybdenum in plants. Acta Biologica Hungarica, 2007, 8, 311-324.
4. Wang Y H, Wei W X, Tan Q L, Du Y Q, Xu S L. Studies on Mo efficiency and application in winter wheat cultivated in yellow brown earth in Hubei. Soils and Fertilizers, 1995, 8, 24. (in Chinese)
5. Kaiser B N, Gridley K L, Brady J N, Phillips T, Tyerman S D. The role of molybdenum in agricultural plant production. Annals of Botany, 2005, 96, 745-754.
6. Lipestt J,, Simpson J R. Analysis of the wheat response by wheat to application of molybdenum in relation to nitrogen status. Aust J Inst Agric Anim Husb,, 1973, 13, 563-566.
7. Du Y Q, Wang Y H, Wei W X, Wang Z W. Effect of molybdenum on molybdenum and nitrogen nutrition in winter wheat. Journal of Huazhong Agricultural University, 1994, 13, 384-389. (in Chinese)
8. Hu C X, Wang Y H, Wei W X, Chen H. Influence of different available molybdenum of yellow brown earth on yield frame, chlorophyll and soluble sugar. Soils and Fertilizers, 1998, 1, 19-22. (in Chinese)
9. Yu M, Hu C X, Wang Y H. Effects of molybdenum on the intermediates of chlorophyll biosynthesis in winter wheat cultivars under low temperature. Agricultural Sciences in China, 2006, 5, 670-677.
10. Yaneva I A, Baydanova V D, Vunkova-Radeva R V. Nitrate reductase activation state in leaves of molybdenum-deficient winter wheat. Journal of Plant Physiology, 2000, 157, 495-501.
11. Shinde P H, Kale S P, More D A, Zende G K. Effect of soil and foliar application of molybdenum on yield of four varieties of wheat. Journal of Maharashtra Agricultural Universities, 1980, 140-142.
12. Hu C X, Wang Y H, Wei W X. Effect of molybdenum applications on concentrations of free amino acids in winter wheat at different growth stages. Journal of Plant Nutrition, 2002, 25,1487-1499.
13. Tiwari K N, Sulewski G, Portch S. Challenges of meeting nutrient needs in organic farming. Indian Journal of Fertilizers, 2005, 1(4): 41-48, 51-59.
14. Cui Z L, Chen X P, Zhang F S, et al. Analysis on fertilizer applied and the central factors influencing grain yield of wheat in the northern China plain. Acta Agriculturae Boreali-Sinica, 2008, 23(S1): 224-229.
15. Liang E, Wang X B, Cai D Y, et al. Influence of reduced fertilization on growth of dryland corn and soil nutrition. Chinese Journal of Agrometeorology, 2007, 28(4): 371-373.
16. Zhang F S, Wang J Q, Zhang W F, et al. Nutrient use efficiencies of major cereal crops in China and measures for improvement. Acta Pedologic Sinica, 2008, 45(5): 915-924.
17. Zhang B Q, Zhang X Q, Wang Q, Li W Y, Huang M, Zhou Q, Cai J, Wang X, Cao W X Dai T B, Jiang D. Increasing plant density improves grain yield, protein quality and nitrogen agronomic efficiency of soft wheat cultivars with reduced nitrogen rate. Field Crops Research, 2021, 267, 108145.
18. Gu X B, Cai H J, Chen P P, Li Y P, Fang H, Li Y N. Ridge-furrow film mulching improves water and nitrogen use efficiencies under reduced irrigation and nitrogen applications in wheat field. Field Crops Research, 2021, 270, 108214.
19. Xia H Y, Li X J, Qiao Y T, Xue Y H, Yan W, Ma L, Zhao Q Y, Kong L G, Xue Y F, Cui Z L, Werf W. Dissecting the relationship between yield and mineral nutriome of wheat grains in double cropping as affected by preceding crops and nitrogen application. Field Crops Research, 2023, 293, 108845.
20. Peng C J, Zhang Z C, Li Y, Zhang Y, Dong H B, Fang Y H, Han L P, Xu W G, Hu L. Genetic improvement analysis of nitrogen uptake, utilization, translocation, and distribution in Chinese wheat in Henan Province. Field Crops Research, 2022, 277, 108406.
21. Huang N, Wang Z H, Wang L, Ma Q X, Zhang Y Y, Zhang X X, Wang R. Yield variation of winter wheat and its relationship to yield components, NPK uptake and utilization of leading and high yielding wheat cultivars in main wheat production regions of China. Scientia Agricultura Sinica, 2020, 53(1): 81-93.

22. Guo, Y, Yin W, Hu F, Fan Z, Fan H, Zhao C, Yu A, Chai Q, Coulter J A. Reduced irrigation and nitrogen coupled with no-tillage and plastic mulching increase wheat yield in maize-wheat rotation in an arid region. Field Crop. Res. 2019, 243, 107615.
23. Anas M, Liao F, Verma K K, Sarwar M A, Mahmood A, Chen Z L, Li Q, Zeng X P, Liu Y, Li Y R. Fate of nitrogen in agriculture and environment: Agronomic, eco-physiological and molecular approaches to improve nitrogen use efficiency. Biol. Res, 2020, 53, 47.
24. Albornoz F. Crop responses to nitrogen overfertilization: A review. Sci. Hortic, 2016, 205, 79-83.
25. Yaneva I A, Baydanova V D, Vunkova-Radeva R V. Nitrate reductase activation state in leaves of molybdenum-deficient winter wheat. Journal of Plant Physiology, 2000, 157(5), 495-501.
26. Imran M, Sun X, Hussain S, Ali U, Rana M S, Rasul F, Saleem M H, Moussa M G, Bhantana P, Afzal J. Molybdenum-Induced Effects on Nitrogen Metabolism Enzymes and Elemental Profile of Winter Wheat (Triticum aestivum L.) Under Different Nitrogen Sources. Int. J. Mol. Sci. 2019, 20, 3009.
27. Yu M, Hu C X, Wang Y H. 1999. Influences of seed molybdenum and molybdenum application on nitrate reductase activities, shoot dry matter and grain yields of winter wheat cultivars. Journal of Plant Nutrition, 21, 1433-1441.
28. Wen X. (2019). Research on the nitrogen transformation in rhizosphere of winter wheat (triticum aestivum) under molybdenum addition. Environmental Science and Pollution Research, 26(3).
29. Liu C, Hu C, Tan Q, Sun X, Wu S, Zhao X (2019). Co-application of molybdenum and zinc increases grain yield and photosynthetic efficiency of wheat leaves. Plant, Soil and Environment, 65(10).
30. Ma Q, Wang M Y, Zheng G L, Yao Y, Tao R R, Zhu M, Ding J F, Li C Y, Guo W S, Zhu X K. Twice-split application of controlled-release nitrogen fertilizer met the nitrogen demand of winter wheat, Field Crops Research, 2021, 267, 108163.
31. Lawes R, Chen C, Whish J, et al. Applying more nitrogen is not always sufficient to address dryland wheat yield gaps in Australia[J]. Field Crops Research, 2021, 262: 108033.
32. Tian Z W, Liu X X, Gu S L, Yu J H, Zhang L, Zhang W W, Jiang D, Cao W X, Dai T B. Postponed and reduced basal nitrogen application improves nitrogen use efficiency and plant growth of winter wheat[J]. Journal of Integrative Agriculture, 2018, 17(12): 2648-2661.
33. Zoz T, Steiner F, Testa J, Seidel E P, Zoz A. Foliar fertilization with molybdenum in wheat. Semina Ciencias Agrarias, 2012, 33(2), 633-638.
34. Imran M, Hu C, Hussain S, Rana M S, Sun X. Molybdenum-induced effects on photosynthetic efficacy of winter wheat (triticum aestivum l.) under different nitrogen sources are associated with nitrogen assimilation. Plant Physiology and Biochemistry, 2019, 141, 154-163.
35. Yu M , Hu C , Wang Y . Influences of seed molybdenum and molybdenum application on nitrate reductase activity, shoot dry matter, and grain yields of winter wheat cultivars[J]. Journal of Plant Nutrition, 1999, 22(9), 1433-1441.
36. Ali S, Hafeez A, Ma X, Tung S A, Chattha M S, Shah A N, Luo D, Ahmad S, Liu J, Yang G. Equal potassium-nitrogen ratio regulated the nitrogen metabolism and yield of high-density late-planted cotton (Gossypium hirsutum L.) in Yangtze River valley of China. Ind. Crop. Prod. 2018, 129, 231–241.

Disclaimer/Publisher's Note: The statements, opinions and data contained in all publications are solely those of the individual author(s) and contributor(s) and not of MDPI and/or the editor(s). MDPI and/or the editor(s) disclaim responsibility for any injury to people or property resulting from any ideas, methods, instructions or products referred to in the content.

Table 1. Total dry weight at maturity, nitrogen accumulation, and NUE relative to N and Mo application rate.

	**Treatment**	**TDW**	**NAE**	**NRE**	**PEPN**	**N accumulation**
		(kg ha^{-1})	(kg/kg)	(%)	(kg/kg)	(kg ha^{-1})
JM44	N0+Mo1	4821.2 i				57.6 d
	N0+Mo2	5228.8 h				57.6 d
	N0+Mo3	6339.2 g				57.6 d
	N1+Mo1	9969.6 f	30.2 a	65.3 a	77.8 c	106.5 c
	N1+Mo2	10837.6 e	28.4 b	70.2 a	83.3 b	110.3 c

	N1+Mo3	12615 b	25.9 c	66.0 a	85.3 a	107.1 c
	N2+Mo1	11917.8 d	17.6 d	62.9 a	41.4 f	152.0 ab
	N2+Mo2	12265.2 c	15.4 e	59.1 a	42.9 e	146.1 b
	N2+Mo3	13655.3 a	14.7 e	66.1 a	44.3 d	156.7 a
SN23	N0+Mo1	5453.0 i				64.5 d
	N0+Mo2	5722.0 h				65.2 d
	N0+Mo3	6207.4 g				63.8 d
	N1+Mo1	10791.1 f	36.3 a	81.1 b	80.2 c	125.3 c
	N1+Mo2	12534.4 e	32.0 b	89.4 a	85.0 b	132.2 b
	N1+Mo3	14041.7 b	30.5 c	86.0 ab	88.4 a	128.3 c
	N2+Mo1	13690.2 c	21.9 d	65.3 c	43.9 d	162.4 a
	N2+Mo2	13412.3 d	17.5 e	62.8 c	44.1 d	159.4 a
	N2+Mo3	15235.0 a	16.5 f	64.7 c	45.5 d	160.9 a
ANOVA	Variety (V)	35263.1**	1692.6**	63.6**	804.6**	638.5**
	N rate (N)	714992.0**	20828.8**	106.0**	309007.8**	12948.2**
	Mo rate (M)	41154.6**	871.3**	ns	1697.9**	ns
	N*V	4219.2**	116.6**	45.0**	32.5**	76.2**
	M*V	69.2**	65.0**	ns	18.7**	4.9*
	N*M	3664.8**	13.8**	5.9**	554.5**	15.4**
	N*M*V	801.0**	ns	ns	17.7**	3.3*

Different lowercase letters within columns indicate significant differences at P < 0.05 across N and Mo rates (n=3). Different lowercase letters within columns indicate significant differences among varieties at P < 0.05 (n=3). *P < 0.05. **P < 0.01. ns, not significant.

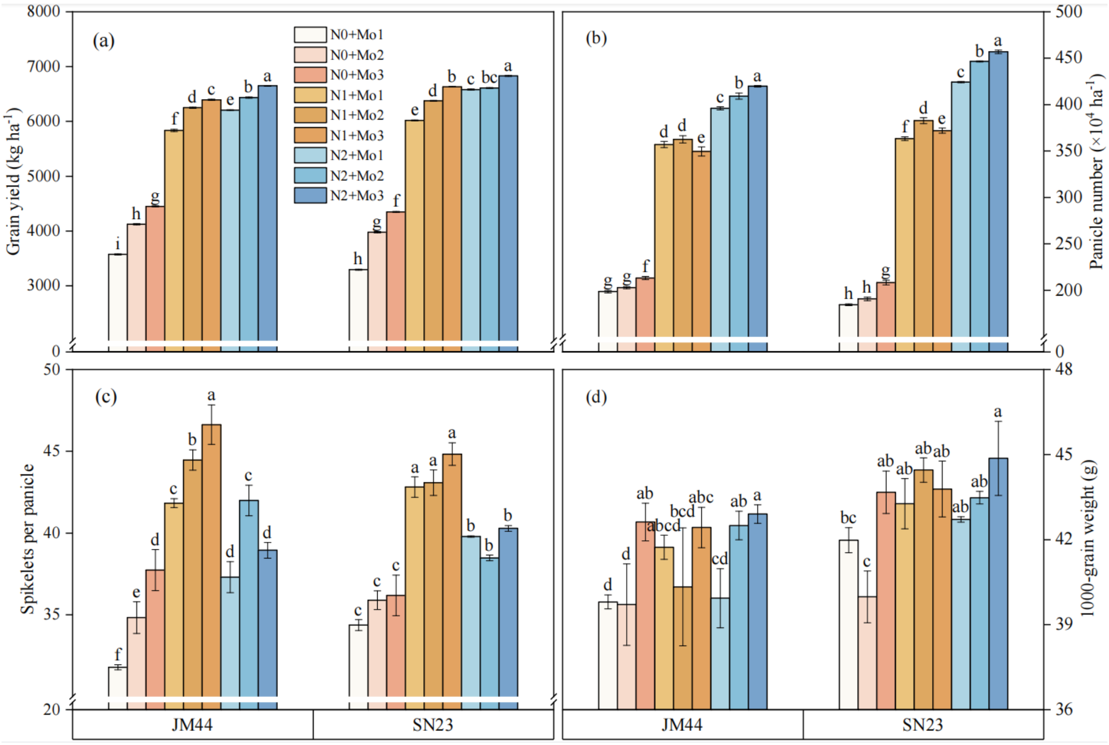

Figure 1. Wheat yield and its components under different N and Mo application levels. (n=3)

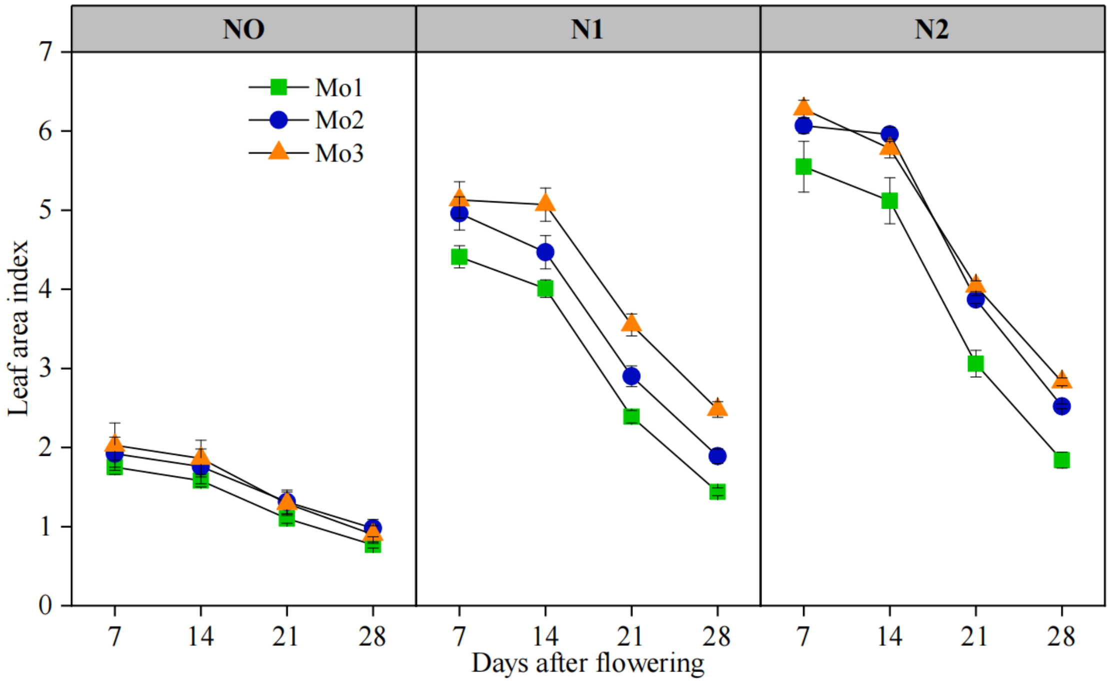

Figure 2. The variation of leaf area index after flowering under different N and Mo application levels. (n=6)

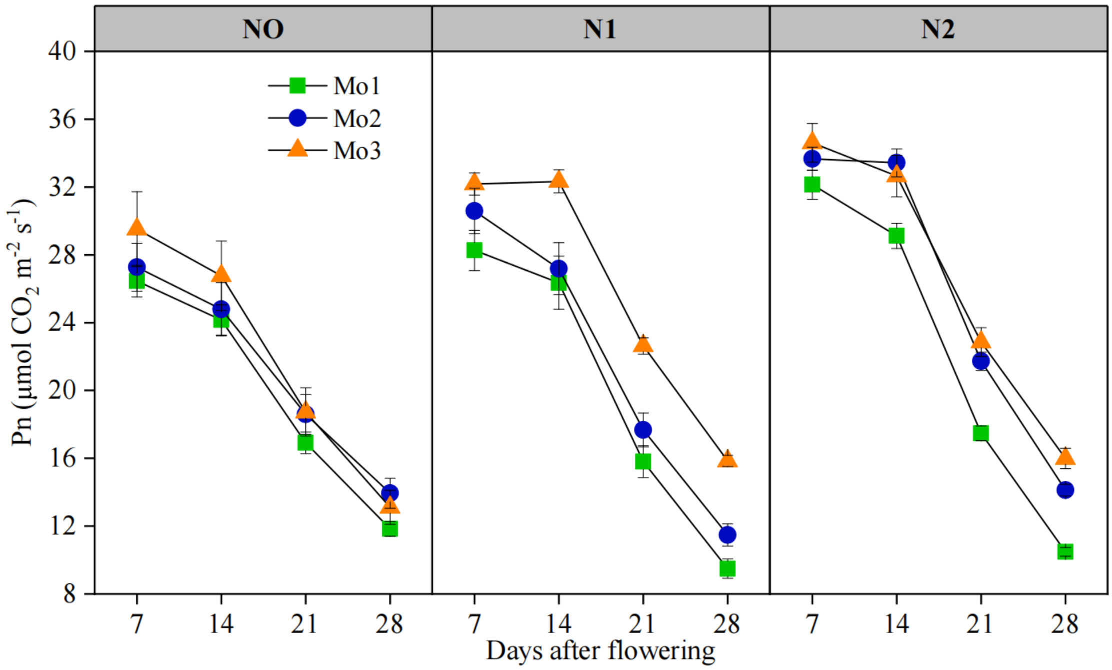

Figure 3. The variation of photosynthetic rate after flowering under different N and Mo application levels. (n=6)

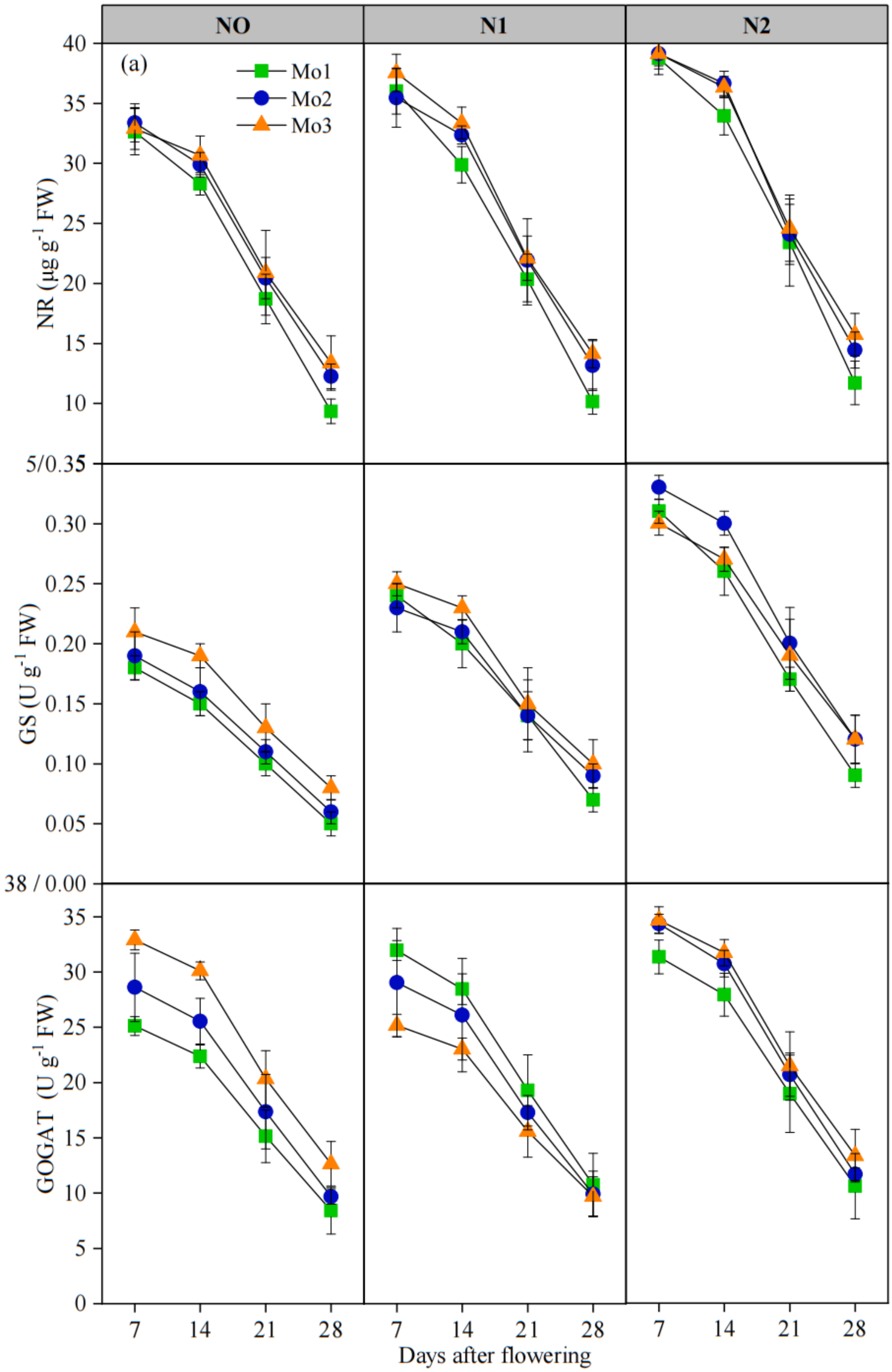

Figure 4. The variation of N-metabolizing enzymes after flowering under different N and Mo application levels. (n=6)

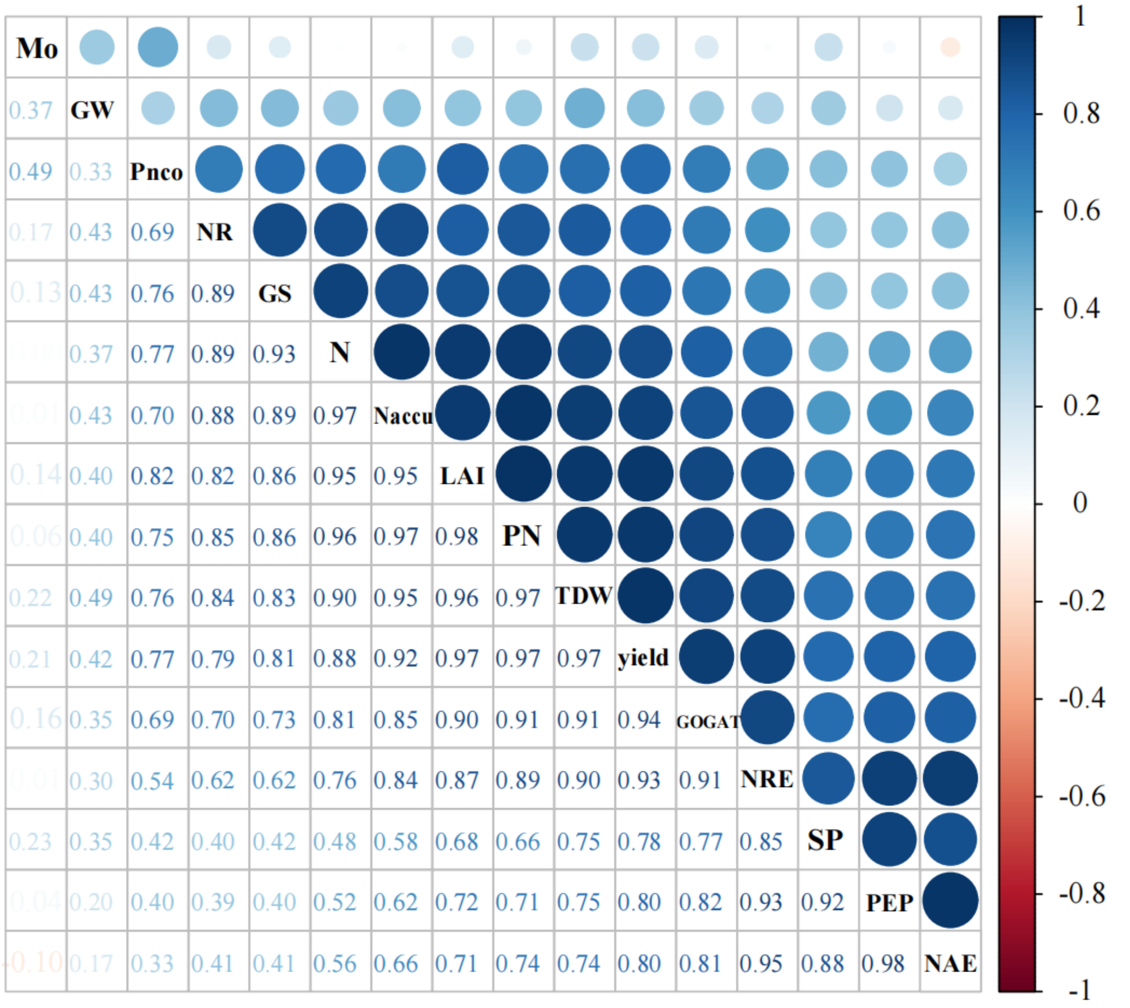

Figure 5. Correlation matrix of various grain yield and population growth parameters (n=54). N, nitrogen application rate; Mo, Mo application rate; PN, panicles number m-2; SP, spikelets per panicle; GW, 1,000-grain weight; TDW, total dry matter; Naccu, nitrogen accumulation; NR, nitrate reductase; GS, gutamine synthetase; GOGAT, NADH-glutamate synthase. Numbers are determination coefficients. *P < 0.05. **P < 0.01.
