# Supplementary material for: Effects of increasing molybdenum under low nitrogen input on yield, nitrogen-metabolizing enzymes and nitrogen use efficiency of winter wheat cultivars
Source: PLoS One. 2026 Mar 5;21(3):e0339840. doi: 10.1371/journal.pone.0339840 (PMC12962463; doi:10.1371/journal.pone.0339840)
Supplement: Attachment — Submitted filename: Reviewer #4.docx [file pone.0339840.s004.docx]

**Responses to reviewer comments:**

Reviewer #4 – Comment 1:

“In line 13 the effects”

Response:

We thank the reviewer for noting this issue. The word “the effects” was incorrectly phrased and has now been corrected for clarity in the Abstract.

Reviewer #4 – Comment 2:

“In line 14 It should be corrected to Se instead of Mo”

Response:

We appreciate this observation. Upon reviewing the Abstract, we confirm that “Se” was mistakenly written instead of “Mo.” It has now been corrected to “Mo” to ensure consistency throughout the manuscript.

Reviewer #4 – Comment 3:

“In line 77 (20-22) There is no reference number 21.”

Response:

Thank you for pointing this out. The citation numbering has been carefully checked, and the missing reference has been corrected to maintain sequential accuracy.

Reviewer #4 – Comment 4:

“In line 89 It should be corrected to field experiments instead of pot experiments”

Response:

We appreciate this clarification. The study was indeed conducted under pot experimental conditions, not field conditions. To avoid misinterpretation, we retained “pot experiments” in Methods but corrected earlier wording in the Introduction to explicitly state that the study used pot experiments.

Reviewer #4 – Comment 5:

“In line 90 plant densities There is no experiments plan with plant density in the article.”

Response:

We thank the reviewer for this observation. The phrase “planting densities” was included in error. Since no planting density treatments were tested, we have removed this wording to avoid confusion.

Reviewer #4 – Comment 6:

“In line 98 2022 instead 2021”

Response:

We agree. The experiment was conducted in 2022, not 2021. This has been corrected in the Methods section.

Reviewer #4 – Comment 7:

“In line 163 FeCI3 (The number 3 should be written as a subscript)”

Response:

We thank the reviewer for this correction. The chemical formula has been corrected to FeCl₃.

Reviewer #4 – Comment 8:

“In line 168 HCl”

Response:

We appreciate this correction. The abbreviation has been standardized to “HCl.”

Reviewer #4 – Comment 9:

“In line 210 ve 216 Table 2 instead of table 1”

Response:

We thank the reviewer for identifying this mislabeling. The in-text references have been corrected to “Table 1.”
